# Development and Evaluation of a Combined Cultivator and Band Sprayer with a Row-Centering RTK-GPS Guidance System

**DOI:** 10.3390/s130303313

**Published:** 2013-03-11

**Authors:** Manuel Perez-Ruiz, Jacob Carballido, Juan Agüera, Antonio Rodríguez-Lizana

**Affiliations:** 1 Aerospace Engineering and Fluids Mechanics Department, University of Sevilla, Ctra. Sevilla-Utrera km 1, 41013 Sevilla, Spain; E-Mail: arodriguez2@us.es; 2 Rural Engineering Department, University of Córdoba, Campus de Rabanales, Edif. Leonardo da Vinci, Ctra. Nacional IV-A km 376, 14014 Córdoba, Spain; E-Mails: jacob.carballido@gmail.com (J.C.); jaguera@uco.es (J.A.)

**Keywords:** weed control, automation, GPS, sugar beet

## Abstract

Typically, low-pressure sprayers are used to uniformly apply pre- and post-emergent herbicides to control weeds in crop rows. An innovative machine for weed control in inter-row and intra-row areas, with a unique combination of inter-row cultivation tooling and intra-row band spraying for six rows and an electro-hydraulic side-shift frame controlled by a GPS system, was developed and evaluated. Two weed management strategies were tested in the field trials: broadcast spraying (the conventional method) and band spraying with mechanical weed control using RTK-GPS (the experimental method). This approach enabled the comparison between treatments from the perspective of cost savings and efficacy in weed control for a sugar beet crop. During the 2010–2011 season, the herbicide application rate (112 L ha^−1^) of the experimental method was approximately 50% of the conventional method, and thus a significant reduction in the operating costs of weed management was achieved. A comparison of the 0.2-trimmed means of weed population post-treatment showed that the treatments achieved similar weed control rates at each weed survey date. Sugar beet yields were similar with both methods (*p* = 0.92). The use of the experimental equipment is cost-effective on ≥20 ha of crops. These initial results show good potential for reducing herbicide application in the Spanish beet industry.

## 1. Introduction

Weeds compete with crops for nutrients, water and light and may reduce yield significantly, especially during early growth, and impair crop quality, resulting in financial losses to the farmer [1,2]. Typically, the selection of a weed control method is determined based on crop variety and condition, weed type and size and available equipment [3]. Chemical methods are frequently used because they control a broad spectrum of weed species. However, precision and automation in weed control technology development have been motivated by increased consumer demand for organic produce as well as consumer and regulatory demands reducing the environmental degradation caused by excessive pesticide and fertilizer usage. Farmers have also experienced a decrease in the availability of workers willing to perform manual tasks such as hand weeding. Alternatives have been developed to reduce or eliminate herbicide applications, a step that is required for organic production [4–7].

The past decade has experienced significant improvements in cultivators and band sprayers that have increased agricultural efficiency. These improvements include steer-by-wire technology linked to global navigation satellite systems (GNSS) utilizing remotely received maps [8,9]. New technologies, such as automated control and robotic sprayers [10], provide opportunities to pursue a different approach for achieving higher productivity while lowering production cost.

The three areas requiring within a typical field are as follows: between rows (inter-row), between crop plants (intra-row) and close (30–40 mm) to the plants [11]. Weeds present between crop rows can be controlled effectively with conventional inter-row cultivation, such as with disc cultivators, brush weeders, rotary hoes, rolling cultivators and rolling harrows [12,13]. Hand hoeing can be eliminated with mechanical weeding in this area. Intra-row weeds are more difficult to eliminate, as they grow within the seed-line [14,15]. Hand labor for intra-row weed removal, band spraying on the seed-line [16,17] and broadcast applications over the whole field are the common practices [18] in sugar beet fields. Countries of central and southern Europe routinely use pre-emergence and several post-emergence herbicide applications with a mixture of many active ingredients. However, mechanical intra-row weeding and manual labor are used when chemical treatments are not effective in treating herbicide-resistant weeds [19].

Genetically modified herbicide-tolerant crops can reduce operational costs [18]. However, despite the use of transgenic organisms in several countries, such as the USA, Canada and Japan, they are not used in regions such as the European Union, Mexico, South Korea, Australia, New Zealand, Colombia, Russia and China [20]. For this reason, in these areas effective weed control has been achieved by the use of herbicides [18]. However, environmental concerns motivate the combined use of spraying and tillage, especially when runoff events are problematic [21].

Sugar beet inter-row cultivators hold a number of rigid or vibrating shanks mounted on half sweeps. These sweeps are distributed in gangs suspended from a toolbar. These cultivators generally cannot work close to the crop plant unless an implement-positioning control system is utilized. Manual implement steering by a second operator has been a common guidance method to control the toolbar position to reduce crop damage by increasing cultivation accuracy. However, three problematic issues remain: increased operation costs, low availability of trained workers and low efficiencies associated with human error, especially during conditions of poor visibility (e.g., at night or in dusty conditions). Hydraulically powered implement systems based on computer vision and GPS guidance technology have been developed to reduce the error caused by the tractor driver [22,23].

Real-time kinematic GPS (RTK-GPS) provides a row-positioning accuracy of ±25 mm, comparable to machine vision guidance systems but without the need for visual guidance landmarks in the field [24]. Targets may not always be visible, such as when the crop has not emerged or is too small. This level of geo-positioning accuracy in row crops can enhance the precision of chemical placement in narrow bands or cultivation close to the plant line [25]. However, one disadvantage of RTK-GPS solutions is the high capital cost due to the requirement that a base station be located within 10 km at all times. GPS service providers and government institutions are working to mitigate this challenge by developing networks of base stations that provide access to RTK correction signals over a wider geographic region via cellular or radio modems or satellites [26].

The overall objective of the present work was to develop and evaluate the performance of an implement suitable for commercial production that combined a row crop cultivator with a band sprayer. This hardware consisted of a retro-fitted row-centering position implement controlled by an RTK-GPS geo-positioning system. The specific objectives were to: (i) design and build a fully automatic electro-hydraulic side-shift frame controlled by GPS location information; (ii) incorporate mechanical inter-row cultivation and intra-row band-spray weed control; and (iii) assess the field performance, weed control efficacy and cost-effectiveness of the combined weeding system compared to conventional systems.

## 2. Materials and Methods

### 2.1. Equipment Design and Manufacture

A system was developed for weed control of inter-row and intra-row areas with a unique combination of cultivation for six rows, a narrow band sprayer and an electro-hydraulic side-shift frame for row center positioning:

#### Side-Shift Frame System

A side-shifting frame was developed for centering the narrow band treatments of herbicide above the rows and parallel to the crop rows with a minimum of lateral drift (cross-track error). For applications with significant side-slope and/or with very wide implements, precise weed control can be best achieved if the implement is also controlled in addition to the tractor navigation.

**Figure 1.**
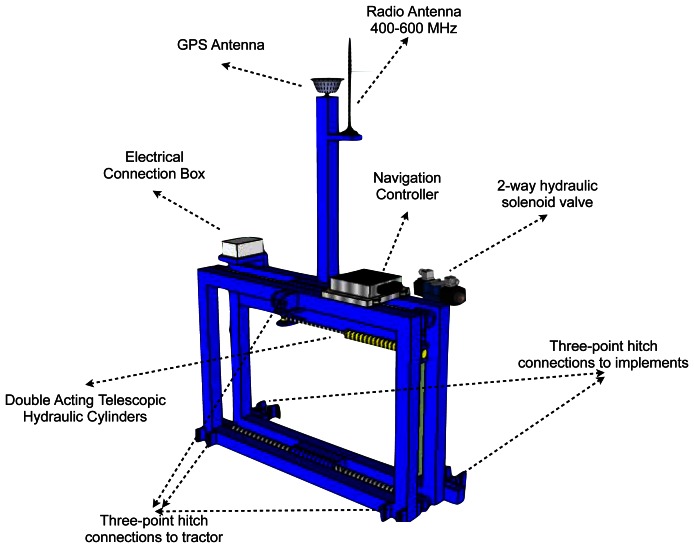
Schematic diagram showing the side-shift frame system developed for row position centering controlled by an RTK-GPS geo-positioning system.

A double-acting hydraulic cylinder with a stroke length of 0.3 m was mounted on the metal frame. This cylinder consisted of a rectangular tube 0.6 m long that was strong enough to support the mechanical and chemical weeding implement (Figure 1). A 2-way hydraulic solenoid valve (model 450–500 psi, Parker Hannifin Co., Cleveland, OH, USA) allowed left/right shifting, and a manual proportional control valve regulated the oil flow rate to vary the piston velocity. A direction-specific calibration setting was used to ensure the same piston speeds for left and right movements.

**Figure 2.**
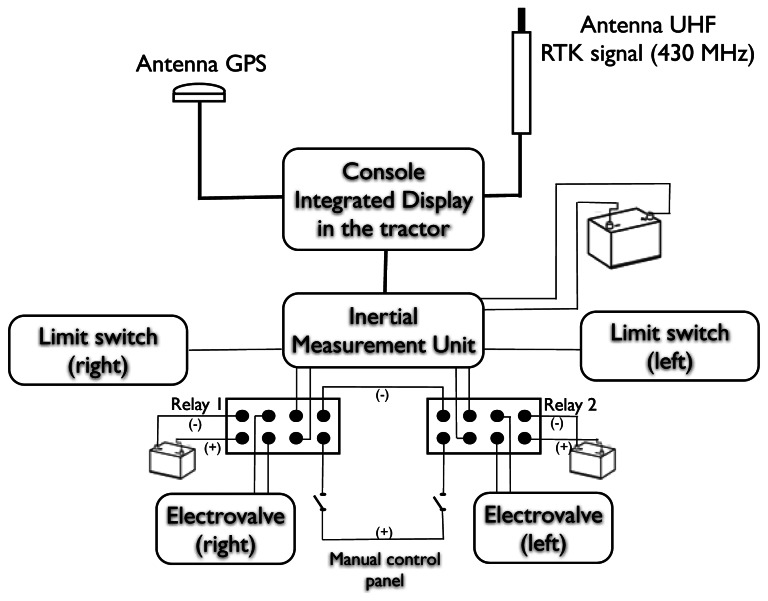
Communication and control diagram for the side-shift frame system.

A positioning sensor was interfaced to a relay control circuit that actuated the hydraulic system on the shifting frame. The controller's function was to operate the 2-way solenoid valve responsible for shifting the frame in one direction. The controller was not connected directly to the valves but was connected to separate 12 V relays responsible for operating the valve (Figure 2). These relays allowed the use of an external control device to manually control the lateral movement of the shifting frame. Two limit switches were used to restrict the motion of the hydraulic cylinder. The side-shift frame was attached to the tractor using the rear three-point linkage.

#### Mechanical and Chemical Weed Control System

**Figure 3.**
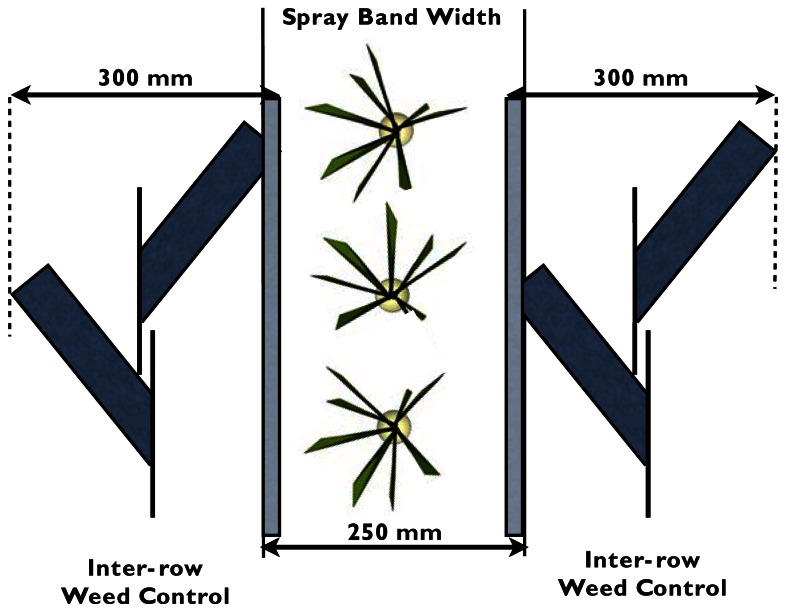
Mechanical inter-row weed control and herbicide spray band with the overlapped zones (gray).

An implement that incorporated tools for mechanical and chemical weeding was attached to the side-shift frame using an anchoring plate. For the inter-row weed control system, seven units were used to cultivate six crop rows. Five central units, consisting of two beet hoes and outer two units, had only one hoe, were mounted on spring shanks and were attached to the implement chassis with an angle plate (90°). The beet hoe shape was selected to provide good cutting performance for both plant material and the high clay soil present on the farm [27]. Figure 3 shows how a set of beet hoes worked between crop rows, 100 mm from the center of the row and with a working width of 300 mm. There was a 25 mm overlap between the spray band and the beet hoes on each side to avoid untreated areas. The system had two gauge wheels for controlling the working depth and two folding bars, joined by hinges at both the left and right sides, to allow a larger implement width that was easily compacted for safer field-to-field transportation.

The hydraulic sprayer components needed to apply herbicide to six crop rows in narrow bands and in broadcast application were mounted on the chassis along with a 500 L tank. In the banded application, the angle of the spray pattern and mounting height of the nozzle were critical in controlling band width. Before field tests, a specific band width was selected and checked with the appropriate nozzle height for a spray angle of 80° with respect to the crop (band width 250 mm and nozzle height 150 mm). In the broadcast application, the nozzles were positioned 500 mm above the crop and separated by 500 mm with a spray angle of 110°, which is the conventional practice of local sugar beet producers.

An initial test of the system was conducted to characterize the lateral implement movement, with a forward speed of 7.5 km/h. A rigid disc was attached to one beet hoe to create a small furrow to indicate the beet hoe path as it passed across the field. A hand ruler was used to characterize the lateral implement movement by measuring the ground distances between this furrow and the crop rows; a similar procedure was described and used by Griepentrog *et al.* [23] The side-shift RTK GPS output string was also logged.

### 2.2. Global Positioning System (GPS)

An RTK-GPS system was used to correct the lateral deviation of the combined row crop cultivator and band sprayer implement. The system consisted of a rover RTK GPS (model AgGPS 450, Trimble Navigation Ltd., Sunnyvale, CA, USA) with the GPS antenna mounted 2 m above the ground and located in the center of the three-point hitch support frame (Figure 1). The system received RTK-fixed quality correction signals from a dedicated RTK base station located ∼0.5 km from the test site. The base station was configured to broadcast compact measurement record (CMR)-RTK correction signals when transmitting through a radio-modem to the RTK receiver mounted above the tractor. The controller (Trimble AgGPS NavController II) was mounted on the side-shift frame 0.8 m below the GPS antenna to compensate for tilt and yaw and provide precise lateral correction information to the implement by using guidance information from the console (model FMX, Trimble) with an internal RTK receiver. The horizontal position dilution of precision (HDOP) was recorded during the field test, and these values ranged between 2.7 and 2.9, indicating that the satellites were well distributed and the computed position was accurate. An RTK-GPS automatic guidance system (AgGPS Autopilot, Trimble Navigation Ltd.) was used to pilot the tractor (John Deere model 6820, John Deere, Moline, IL, USA) during the seeder operation. The AB line used for seeding was stored internally in the tractor navigation system for future use during the weed control trials.

### 2.3. Field Experiments

Large-scale field tests were conducted during the 2010–2011 sugar beet season in the Sevilla region located in the southern part of Spain (36.95436°N, 6.084717°W). Approximately 8 ha were planted with a 12-row pneumatic drill seeder in a commercial sugar beet field, within which a 1 ha section was selected for the weed control trials. The tractor used for the seeding operations was guided with an automatic steering system of centimeter-level precision to ensure straight seed-lines and to generate a 6 m AB line, which was converted into two 3 m AB lines for use during the trials. A 3 m offset distance was added using the user-interface of the automatic steering system; this distance is a typical implement width (here, the experimental implement width was 3 m).

In this experimental plot, two types of weed control treatment (*i.e.*, conventional and experimental herbicide application) were compared to analyze the herbicide savings and efficiency achieved when a side-shift frame based on the RTK-GPS correction was used for weed control. Both treatments were performed at a rate of 225 L ha^−1^, 4 bars of pressure and a nominal tractor speed of 7.5 km h^−1^. The tractor (Kubota model B2530, Torrance, CA, USA) used for the test was a small tractor of 18 kW rated power. This light-weight tractor has tractive characteristics that allow field entry only a few hours after rain, which is an important consideration in this area of marshy fields.

Conventional or broadcast herbicide applications were conducted on six experimental plots, applied uniformly on the ground (pre-emergence) or over the crop canopy (post-emergence), and the experimental applications were conducted on six experimental plots. Each of these experimental plots was 216 m^2^, and 15 untreated control plots with a total area of 18 m^2^ were left between these plots. The treatments were randomized between different experimental plots.

One pre-emergence and three post-emergence herbicide applications were carried out in this test. For the conventional application, the shifting frame was not activated; thus, the spraying operation resembled the common practice of local farmers. The 6 nozzles were set at 50 cm above the crop and 50 cm between nozzles, with a spray angle of 110°. For the experimental weed control treatment, the automatic shifting frame with RTK-GPS-based control was used to correct the implement position, and the banded spray was set to 6 nozzles located 150 mm above the crop and separated by 500 mm with a spray angle of 80°. The wetted surface using the conventional application was 3 m, and the surface using the banded application was 1.5 m. The mechanical cultivation tools were not required until the third post-emergence herbicide treatment because until then, the weeds between rows were not in competition with the crop.

Ten days after this 3rd treatment, the weeds that had escaped the mechanical/chemical control were removed manually. The time spent by the tractor driver and the hand-hoeing crew were recorded to calculate weed control costs. To assess the impact of the two methods on yield, the sugar beets in the experimental trials were harvested, and Wyse's method [28] was used to correct to a standard 16% sugar content.

### 2.4. Data Analysis

#### Determination of the Cross-Track Error

In many agricultural applications, such as tillage, planting, spraying and harvesting, the vehicle passes should be parallel and separated by a constant distance H. If the actual distance is greater than H, an area can be skipped, and if the actual distance is less than H, there is an overlap.

The single-point cross-track error (XTE_i_) was defined as the perpendicular distance from the straight line AB to each recorded RTK GPS system point. The total XTE was calculated using the RMS value of all the single-point XTEs along the full length of the line AB [29]. Cross-track error is an important variable that affects the potential skip or overlap. The real distance can be calculated from the simple analytic geometry shown in Equation (1):

(1) $$ɛ{\text{RMS}}_{t}=\sqrt{\frac{1}{N_{t}}\sum_{i=1}^{N_{t}}e_{it}^{2}}$$

For each pass, a root mean squared (RMS) error was then calculated with the following equation:

(2) $${e^{2}}_{it}={\left(x_{i}-x_{t}\right)}^{2}+{\left(y_{i}-y_{t}\right)}^{2}$$

where:

RMS_t_ = the RMS error for the *t*th pass
N_t_ = total number of measurement point for the *t*th pass
e_it_ = distance from the *i*th point to the *t*th pass
Descriptive statistics and the Shapiro-Wilk contrast were calculated using R software [30].

#### Weed Control Efficacy Study and Crop Yield Response

In the untreated control, conventional and experimental plots, weed counts were used to evaluate the weed population (plants m^−2^). The weed species included knotted hedge parsley (*Torilis nodosa* L.), hairy buttercup (*Ranunculus sardous* L.), scarlet pimpernel (*Anagallis arvensis* L.) and oxtongue (*Picris echioides* L.). Weeds were counted after each post-emergence application (one week) using a rectangular steel frame with a size of 0.5 m × 2 m, and the area under the frame was 5% of the experimental unit size. This area was selected according to the published principles of weed science [31], which recommend that the area under all the quadrats be 5 to 10% of the plot size. The rectangular frame was placed by throwing it into the experimental units where weed infestation was representative of the treatment. The weed population in the control experimental units was used as a reference for all other treatment experimental units.

Statistical analysis of the data was performed with an analysis of variance (ANOVA) model in a completely randomized design with two fixed factors to determine the effects of treatments on the total weed population. The factors were the date of treatment (three levels: 12/02/10, 01/05/11 and 02/22/11) and the treatment type (three levels: conventional application-CA, experimental application-EA and control). The response variable was the population of emerged or surviving weeds after the treatments.

The homogeneity of variances was tested using the Levene test, and normality was tested using the Shapiro-Wilk test. The heteroscedasticity of the surveyed populations led to the use of a robust model [32] using 0.2-trimmed means. Differences between means in the ANOVA models were compared using the Yuen-Welch test [33].

In addition, a comparison of the 0.2-trimmed means of independent populations to determine the effect of CA and EA treatments on crop yield was undertaken. The dependent variable was the average sugar beet yield (t ha^−1^) at standard 16% sugar content. The sugar beet yield was not harvested in the control zone. Analyses were performed with IBM SPSS Statistics 19 and R software [30].

#### Feasibility Study

Engineering economics comparisons require at least two alternative proposals for prospective receipts and disbursements. In this study, we compared the payment required for the purchase of a conventional sprayer or an experimental machine, which are the two alternatives presented for analysis. The difference between payments on investment is 10,200 €.

Cash flow analysis is necessary and was included in this feasibility study [34]. Cash flow analysis includes separate components, such as investment payment (K_o_), or the amount to pay for the implementation of the project; cash flow (F_j_), calculated as the difference between receipts and annual payments; rate of interest (r), according to the expectations of the investor; and the project life (N).

To determine cash flow, we started from the fact (shown in the Results and Discussion section) that crop yield will be similar between methods using each of the two machines. Thus, to determine the increase in cash flow, we only evaluated the difference between payments. This restriction is necessary to evaluate only those differences that are exhibited between the two applications. Table 3 shows the annual cost associated with the use of each alternative. This total included the cost of herbicide application and hand weeding, insurance, GPS-RTK signal fee, fuel, repair and maintenance for both scenarios.

The costs for herbicide application were determined according to the current prices of the ingredients used and the cost of wages in the local area. Furthermore, the GPS signal subscription fee for 1 year and cost of fuel were based entirely on the theoretical fuel consumption [35]. Insurance was obtained by applying a percentage of the purchase price of the equipment, 0.25%, similar to that used by Srivastava *et al.* [36]. Finally, the costs of repairs and maintenance were determined using the following equation:

(3) $$\frac{C_{m}}{P_{u}}=RF1{\left[\frac{t}{1000}\right]}^{RF2}$$

where:

Cm = accumulated repair and maintenance, euros
t = accumulated use, h
RF1, RF2 = repair factors from [36]

The discount rate in the presence of inflation was fixed at 6%, a conservative rate given the current rates in the country. The working lifetime of the sprayer used for economic estimation was 10 years, in accordance with other publications [37].

Frequently, in economic analysis, some parameters are based on assumptions that are difficult or impossible to verify a priori. Therefore, it is common to perform a sensitivity analysis of those parameters that are most likely to be affected by the outcome of the analysis, thus providing simultaneous scenarios that can lead to very different results. This study sensitized inflation cash flows, providing variations between −4% (unfavorable scenario) to +2% (favorable assumptions). We also analyzed scenarios of different areas cultivated by the owner, including 5, 10, 15, 20, 50 and 100 ha. Finally, analyses were carried out with two payments of investment, the initial estimate by the authors, 10,200 €, and another, unfavorable investment of 12,750 €, an increase of 25% above the initial estimate. The life of the machine, always difficult to determine [38], was not included in the analyses because the results have made it unnecessary. This method provided a total of 84 scenarios.

The index used to determine the return on investment was the recovery period (Equation (4)), defined as the year n that *φ* ≥ 0 such that:

(4) $$\left(\varphi =-K_{o}+\sum_{j=1}^{N}\frac{F_{j}}{{\left(1+r\right)}^{j}}\right)$$

This criterion must be satisfied for n ≤ N to guarantee profitability. When considering the inflation rate, the cash flow used to determine the rate of recovery was:

(5) r∗=r−q

where q = annual inflation rate in cash flow.

## 3. Results and Discussion

**Figure 4.**
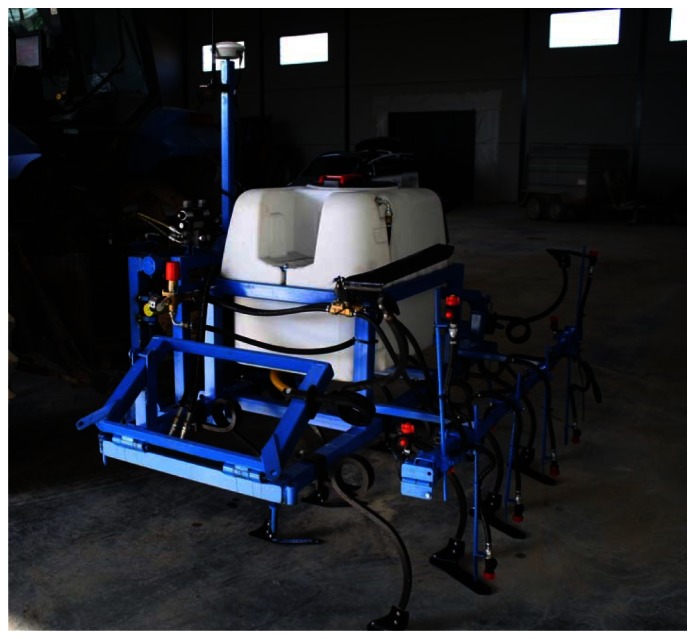
Prototype of six-row mechanical weed control cultivator for inter-row areas and band spraying for intra-row areas.

In this study, an experimental implement, which combined six-row crop cultivators and six band sprayers with row-position centering using an electro-hydraulic side-shift frame, was developed and operated for weed control within inter-row and intra-row areas (Figure 4). The GPS antenna mounting location on the frame and the open nature of the sugar beet field enabled an unobstructed view of the sky during the entire trial. This condition allowed for optimal signal reception regardless of satellite geometry, and RTK GPS fixation was obtained for the recording of all passes during this experiment.

**Figure 5.**
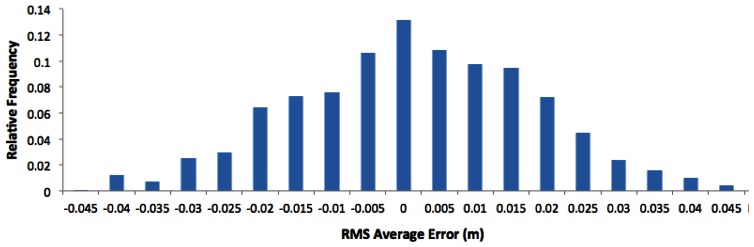
Relative frequency histogram of mean lateral deviations.

A total of 1,409 events were automatically recorded in three different passes. XTE was analyzed, which was the distance between the side-shift frame's actual position and the reference pass at each moment. The GPS receiver error was the transverse deviation from the travel direction. The frequency histogram (Figure 5) shows a good correspondence between the average and median position error, with an asymmetry coefficient of −0.02, and a normal distribution. The Shapiro-Wilk test yielded a p-value of 0.16. The absolute deviation of the modal value was 4 mm, and the 95% transverse deviation was between 0 and ±33 mm at 7.5 km/h. The magnitude of the RMS transverse deviation errors presented a level of accuracy comparable to the results of Griepentrog *et al.* [23] who observed cross-track error mean values of between −16 mm and 11 mm.

All measurements of lateral implement movement, *i.e.*, the ground distances between the mark left by a rigid disc and the crop rows, were located within the intra-row bandwidth, which for this study was defined as ±125 mm from the row center line. This result confirms that the side-shift control did not cause transverse interaction between beet hoe units and the sugar beet plants.

### 3.1. Weed Control Efficacy Study and Crop Yield Response

The weed control efficacies of the treatments (conventional and experimental methods) and the control (no treatment) were compared. Some descriptive statistics are shown in Table 1, including the mean, trimmed means of weed counts, weed surveying dates and sugar beet yield for each treatment. These data illustrate the variability between treatments and dates, with the variance on the earliest date (12/02/10) being consistently greater than on other sampling dates. The data also illustrate that the variability of the weed population was greatest with the conventional treatment on the final date (02/22/11), which, according to AIMCRA technicians, is a key factor in the sugar beet yield. This variability was confirmed by the fact that the conventional treatment exhibited greater yield variations, as observed in the variance estimators and normalized median absolute deviation (Table 1). The experimental treatment tended to provide a more uniform yield by providing a more uniform control of adventitious weeds. The analysis of variance yielded p-values of 0.001 (date factor), 0.003 (treatment factor) and 0.032 (interaction). Therefore, the results were significantly different for all components within the model.

The results indicate that treatments CA and EA achieved similar global weed control for each of the survey dates, with both being significantly better than the control (Table 2). Comparing trimmed means, the control zone showed between 67% and 132% more weeds than those that received conventional or experimental treatments during the crop cycle. No significant differences between treatments CA and EA were observed (Table 2). However, for the first two survey dates, treatment CA had consistently fewer emerging weeds. The difference on 01/05/11 was notable, with 23.6 weeds m^−2^ (CA) *vs.* 34.6 weed m^−2^ (EA). These results were expected. The experimental treatment did not use mechanical weed control between crop rows until the last post-emergence treatment. At this time, weeds between the application bands were controlled with mechanical cultivation. In general, there was a downward trend in the population of weeds in the two treatments studied. This result contrasted with the control treatment, where there was an initial increase in weed population. Thus, the interaction of the model was significant.

As shown in Table 2, on 12/02/10, there was no significant difference between treatments. In weed counts carried out on 01/05/11 and 02/22/11, the plots that received no treatment (control) had a significant increase of weed population. As expected, without weed control, the competitive weed pressure on the crop increased significantly. There were no significant differences between CA and EA in weed count on 02/22/2011.

The data highlight the fact that even though the experimental treatment (EA) only used the mechanical method on 02/22/11, which resulted in a 30% increased weed population with respect to CA (not significant) on 05/01/2011 and 46% (not significant) on 12/02/10, the sugar beet yields from the EA and CA treatments were similar, with nearly identical 0.2-trimmed means (Table 1). There was no significant difference between the two management systems (*p* = 0.92). The sugar beet yield confidence interval for the difference between the 0.2-trimmed means of the CA and EA treatments was (−4.45 t ha^−1^, 4.08 t ha^−1^). This result is consistent with previous indications and the visual field observations of AIMCRA technicians that the weed population at the time of the last herbicide application (with almost identical values between CA and EA) is the most important determinant of sugar beet yield [39,40].

### 3.2. Feasibility Study

Table 3 shows payments to be made with the two machines in different treatments, including through the payment of fuel required by the tractor in operation. All other payments attributable to the tractor (e.g., tires, maintenance, *etc.*) are independent of the machine used. Weed control costs for both treatments were significantly different. The optimized equipment provided a band application width of 250 mm and a nozzle height of 15 cm (80° nozzle). This setting reduced the flow by half, and only half of the ground area received herbicide, but the intra-row plants received the same herbicide dosage. This result implies that the herbicide usage cost, both pre-emergence and post-emergence, could be reduced by 50% compared to conventional treatment. The reduction of the herbicide application rate observed in this study was consistent with the level of reduction observed by Wartenberg and Dammer [40] in a precision spray application using an opto-electronic sensor for weed counts within the tramlines.

Table 3 shows that the payment of hand weeding labor was reduced from 117.96 € ha^−1^ to 101.56 € ha^−1^, a reduction of 14% per hectare, using the experimental system. Fennimore *et al.* [41] reported similar savings in broccoli and lettuce using a machine vision guidance system to control the fine movements of the cultivator. The inter-row tools were used only in the third post-emergence treatment because at the early stages of weed development between the rows, there was negligible risk of competition for nutrients, light and water between the weeds and sugar beet plants. The mechanical weed control applied at the third post-emergence treatment reduced the time spent on hand weeding from 15.32 h ha^−1^ to 13.19 h ha^−1^.

The conventional equipment cost 9,800 €, whereas the experimental equipment had an estimated cost of 20,000 €. The RTK-GPS system, which was still fairly expensive for this practice, was similar in cost to that observed by Pedersen *et al.* [42] who indicated that the price is expected to decrease as the technology becomes more widespread. For these early trials, a dedicated base station was used to transmit the GPS correction signal to the rover receiver. It was possible to achieve an accuracy of ±20–30 mm, but this increased the investment costs of the equipment. Currently, the Andalusian government is working on developing a network of fixed stations. In the future, the GPS correction signals may be used without having to invest in a base station (7,500 €). In this case, the experimental equipment would cost 12,500 €, a significant reduction.

The sensitivity analysis for the investment was generated with seven annual updating rates, two increases of investment payments and six farm sizes, generating 84 possible scenarios (Table 4). Using annual receipts and payments avoids terms that are difficult to quantify, such as depreciation, amortization and interest of fixed or circulating capital [43]. Table 4 shows the payback time in years and indicates that farmers with 5 ha would never recoup their investment using our experimental system. A similar situation is true for farmers with 10 ha, for whom the period of return on investment would be higher than the lifetime of the machine. At these small scales, farmers would need to form an agricultural cooperative association before either system would provide a profitable payback. This experimental equipment has sufficient degrees of freedom to allow adaptation to different row-crop species and plant growth stages.

A single farmer would need more than 15–20 ha to recover his or her investment according to the increase in the payment of investment to consider. Additionally, 15 ha would provide the greatest variation (7 to more than 10 years) in payback recovery time. This farm size would also increase the farmer's risk by relying on low interest rates for shorter payback periods, which neglect the effects of inflation upon annual cash flows. For 20 ha, the system would be profitable even in the most unfavorable conditions of this analysis. A payback time of 6 years for the experimental system would decrease to one year or less for 100 ha farms. This increased cost-effectiveness is mainly due to savings in herbicides, which become the determining variable for the entire term of the analysis. According to these results, the experimental equipment is recommended for areas larger than 20 ha.

Therefore, this study demonstrated the following:

(i) The side-shift frame system developed for row-position centering controlled with an RTK-GPS exhibited similar weed control efficacy as the conventional treatment. The amount of herbicide and the hand-weeding time were reduced, thereby reducing the cost of crop production. Utilization of RTK GPS equipment for other tasks in the crop production system can help disperse the equipment cost across many cultural practices, reducing the equipment cost penalty in the weed control operation and possibly making it economically viable in conventional production systems.
(ii) The sugar beet yields obtained were similar with both application methods (conventional and experimental herbicide application).
(iii) The experimental sprayer is economically profitable for farms above 20 ha of sugar beets according to the simulations.

## 4. Conclusions

A machine that combined six-row crop cultivators and six narrow-band sprayers with row-position centering using an electro-hydraulic side-shift frame controlled by GPS was developed for weed control in both inter-row and intra-row areas, respectively. Field tests showed that the machine was robust, adapting to the working conditions required of this type of implement. The following conclusions were drawn based upon the results of this research:

- The experimental system, equipped with GPS technology, developed and used in this work provided an herbicide band application volume targeted to the crop rows without reducing the quality of the intra-row chemical control treatment and while providing herbicide savings of approximately 50%. These reductions in applied chemicals not only reduce production costs but also reduce the environmental impact caused by the chemicals. Statistical analyses revealed no significant differences with respect to weed control efficacy between the two weed control strategies studied.
- The labor required to hand-weed was 15.3 h ha^−1^ in the conventional treatment and 13.2 h ha^−1^ in the experimental treatment, on average. At prevailing wage rates, the weeding costs were 117.00 € and 101.56 € ha^−1^, respectively. This difference represented a 14% savings with the experimental system.
- Under normal conditions and with the technology used, a farmer with 20 ha using the experimental equipment would be profitable with respect to the conventional equipment, with a payback period of less than the life of the machine. Thus, the experimental equipment can be an affordable option for both large and small farms.
- The adoption of new procedures and technologies that optimize farm operations will help the Spanish sugar beet industry to remain competitive in the global economy.

## Figures and Tables

**Table 1.** Weed population for three survey dates and sugar beet yield statistics.

**Treatment**	**Survey date**	**x̅**	**σ^2^**	**Minimum**	**Maximum**	**0.2-Trimmed mean**	**NMAD ^†^**

		**Weed population (weed m^−2^)**					
CA ^þ^	12/02/2010	35.1	463	3	61	34.5	29.6
	01/05/2011	22.9	36	14	28	23.6	5.0
	02/22/2011	11.4	17	7	17	11.2	5.4
EA ^þþ^	12/02/2010	47.6	540	18	77	47.8	20.0
	01/05/2011	33.4	69	19	44	34.6	4.0
	02/22/2011	10.9	5	8	15	10.6	2.2
Control	12/02/2010	48.9	1340	13	150	39.9	23.7
	01/05/2011	65.6	485	38	108	63.4	25.2
	02/22/2011	47.1	616	15	97	45.6	26.0
	**Yield (t ha^−1^)**						
	
CA ^þ^	07/15/2011	95.4	60.0	75.7	107	95.8	8.5
EA ^þþ^	07/15/2011	96.5	13.8	91.3	106	96.0	2.6

† Normalized median absolute deviation.

þ Conventional application.

þþ Experimental application.

**Table 2.** Comparison of the 0.2-trimmed means of weed population (weeds m^−2^) between treatments and survey dates.

**Treatments (means)**				

**Dates**	**Conventional (CA)**	**Experimental (EA)**	**Control**	**Date factor (mean)**
12/02/2010	34.5 a	47.7 a	39.9 a	39.9 ab
01/05/2011	23.6 a	34.6 a	63.4 b	42.2 a
02/22/2011	11.2 a	10.6 a	45.6 b	23.1 b

Treatment factor (mean)	19.6 a	27.4 a	45.6 b	

Within each factor level, values followed by different letters are significantly different (*p* < 0.005 for both factors).

**Table 3.** Payments for weed control for both applications.

**Area payments**	**Conventional application method**	**Experimental application method**
Herbicide cost (€/ha)		
- Pre-emergence	81.67	40.84
- 1st Post-emergence	38.64	19.32
- 2nd Post-emergence	74.95	37.47
- 3rd Post-emergence	145.83	72.92
Hand weeding (h/ha)	15.32 *	13.19 *
Worker cost (€/ha)	117.96	101.56
Cost of fuel (€/ha)	5.32	6.92
Total cost per ha (€/ha)	459.05	272.11
ANNUAL PAYMENTS		

Insurance (€/year)	147	307.50
Repair and maintenance (€/year)	Variable	Variable
GPS-RTK signal costs (€/year)	0	820
INVESTMENTS		

Electro-hydraulic side-shift frame (€)	0	900
Mechanical and chemical weed control system (€)	0	10,000
RTK-GPS (€)	0	9,100
Conventional broadcast sprayer (€)	9,800	0

* Not summed in these columns because of differences in units (*i.e.*, h/ha and €/ha).

**Table 4.** Payback time (years) for the comparative economic analysis of our experimental system compared to a conventional system.

	**Increase of Investment Payments (€)**											

	**10,200**						**12,750**					

	**Farm size (ha)**											

**r**^✠^(%) ^†^	**5**	**10**	**15**	**20**	**50**	**100**	**5**	**10**	**15**	**20**	**50**	**100**
4	†	>10	7	5	2	1	†	>10	10	6	2	1
5	†	>10	8	5	2	1	†	>10	10	6	2	1
6	†	>10	8	5	2	1	†	>10	>10	6	2	1
7	†	>10	9	5	2	1	†	>10	>10	7	2	1
8	†	>10	9	5	2	1	†	>10	>10	7	2	1
9	†	>10	10	5	2	1	†	>10	>10	7	2	1
10	†	>10	10	6	2	1	†	>10	>10	7	2	1

† Never recovers due to continuous negative cash flow;

✠ Annual updating rate.
